# Knowledge, Attitudes, and Practices Regarding Infection Prevention Among Healthcare Professionals and Their Predictors at a Tertiary Hospital in Ethiopia: A Cross‐Sectional Study

**DOI:** 10.1002/hsr2.71900

**Published:** 2026-03-10

**Authors:** Abebe Dukessa Dubiwak, Mulualem Tadesse, Belay Zewdie, Abebaw Tiruneh, Tadele Akeba Diriba, Selam Tesfaye, Amare Assefa, Gemeda Abebe, Lelisa Sena Dadi

**Affiliations:** ^1^ Department of Epidemiology, Faculty of Public Health Jimma University Jimma Ethiopia; ^2^ Department of Biomedical Sciences, Faculty of Medical Sciences Jimma University Jimma Ethiopia; ^3^ School of Medical Laboratory Sciences, Faculty of Health Sciences Jimma University Jimma Ethiopia; ^4^ Mycobacteriology Research Center, Institute of Health Jimma University Jimma Ethiopia; ^5^ Department of Statistics, College of Natural Sciences Jimma University Jimma Ethiopia; ^6^ Department of Clinical Oncology, Faculty of Medical Sciences Jimma University Jimma Ethiopia

**Keywords:** attitude, control, healthcare professionals, infection, knowledge, practice, prevention

## Abstract

**Background:**

Assessing healthcare professionals' existing knowledge, attitudes, and practices (KAP) regarding infection prevention and control (IPC) is essential for identifying gaps and informing strategies to mitigate healthcare‐associated infections (HCAIs). Thus, the aim of this study was to assess the IPC KAP of healthcare professionals.

**Methods:**

An institution‐based cross‐sectional study was conducted from August to November 2023 among 328 healthcare professionals at Jimma University Medical Center (JUMC). Data were collected using a structured tool.

**Results:**

Overall, 72.3% [95% CI: 67.0%, 77.0%] of participants demonstrated good knowledge, 65.9% [95% CI: 61.0%, 71.0%] had a favorable attitude and 57.0% [95% CI: 52.0%, 62.0%] reported safe IPC practices. The predictors of good knowledge included having IPC training (AOR, 2.31; 95% CI, 1.23, 4.35), availability of IPC guidelines in the department (AOR, 5.87; 95% CI, 2.08, 16.44), and ≥ 10 years of service (AOR, 3.56; 95% CI, 1.28, 9.68). Similarly, having IPC training (AOR, 1.80; 95% CI, 1.08, 2.98), and availability of the IPC guidelines (AOR, 2.14; 95% CI, 1.34, 3.42) were the factors that associated with favorable attitude. Safe IPC practices were predicted by IPC training (AOR, 1.75; 95% CI, 1.06, 2.89), availability of IPC guidelines (AOR, 2.65; 95% CI, 1.62, 4.36), and availability of water and handwashing facilities (AOR, 1.99; 95% CI, 1.24, 3.21).

**Conclusions:**

Despite relatively high level of knowledge and attitude, a considerable proportion of health professions reported unsafe IPC practices. Strengthening IPC training, ensuring guideline availability, and improving infrastructure such as handwashing facilities are crucial to enhance compliance with IPC measures in tertiary care hospitals thereby mitigating HCAIs and AMR.

## 1 Introduction

Modern healthcare supports patient treatment and recovery through invasive devices and procedures; however, it is strongly associated with healthcare‐associated infections (HCAIs) [1]. HCAIs are defined as infections acquired by patients while receiving healthcare services in medical facilities [2]. The most common types include central line‐associated blood stream infections, surgical site infections (SSIs), catheter‐associated urinary tract infections, and ventilator‐associated events [1, 3, 4, 5].

Hospitals provide a conducive environment for reservoirs of wide varieties of microorganisms [6]. Patients are at risk of infections due to compromised health status, underlying medical conditions, or exposure to healthcare interventions such as surgery, diagnostic testing, or invasive devices [7, 8]. Furthermore, hospital settings facilitate microorganism transmission between patients and healthcare providers due to close proximity, frequent contact, and share environments [9, 10].

HCAIs represent a major challenge to healthcare systems, and are the second most prevalent cause of death worldwide [11, 12]. They contribute to considerable morbidity, a prolonged hospital stay, antimicrobial resistance (AMR), long‐term disability, and increased healthcare costs [11, 13]. AMR is closely linked to HCAIs and constitutes a major public health challenge globally [14, 15]. A pooled analysis reported that the prevalence of AMR in HCAIs was 36%, with the risk of death increasing by 58% [14]. Similarly, a study conducted in Lebanon found that multidrug resistance bacterial infections accounted for 71% of SSIs [16], while another Lebanese study reported AMR prevalence as high as 85.7% among female patients with urinary tract infections [17].

The incidences of AMR have further increased during the COVID‐19 pandemic, likely due to poor adherence to antimicrobial stewardships (AMS) programs and misuse or overuse of antimicrobials [18, 19, 20]. The burden of HCAIs as well as AMR is particularly worsened in low‐ and middle‐income countries (LMICs), where inadequate knowledge of infection prevention, poor adherence to universal precautions, and limited access to personal protective equipment (PPE) exacerbate the problem [6, 8, 12]. In line with this, a previous study from Ethiopia highlighted the escalation of AMR in HCAIs during the COVID‐19 pandemic, reporting that 84.37% of ESKAPE pathogens isolated from patients with SSIs were multidrug‐resistant [21].

Effective infection prevention practices are fundamental to ensuring quality of care, and play a critical role in protecting patients, healthcare workers, and communities from HCAIs and AMR [22, 23]. To minimize these risks, healthcare professionals strictly adhere to infection prevention procedures [24, 25, 26]. Inadequate knowledge and poor infection prevention and control (IPC) practices place both patients and providers at risk [27, 28, 29]. Thus, it is imperative that healthcare professionals are equipped with updated, evidence‐based information to effectively implement IPC measures [24, 25, 26]. Any gaps in knowledge or practice contribute to the transmission of microorganisms, accelerate AMR, and hinder preventive efforts [11].

Assessing the knowledge, attitude, and practices (KAP) of healthcare professionals toward IPC is essential to identifying gaps and addressing challenges related to HCAIs, particularly those raising from poor compliance with IPC guidelines [30]. Strengthening KAP and improving adherence to standard IPC practices can significantly reduce HCAIs [12]. Several studies have investigated KAP levels and associated factors among healthcare professionals in Ethiopia [31, 32, 33]. However, previous studies did not account for clustering of responses within professional disciplines, which may lead to underestimated standard errors and spurious statistical significance when using the standard logistic regression model [34, 35]. Accounting for clustering by discipline allows for modeling random effects arising from intracluster response dependence, thereby improving the validity of statistical inference. Hierarchical logistic regression models address these issues and provide more reliable conclusions [34]. Thus, this study aimed to assess the IPC‐related KAP of healthcare professionals and its predictors using a hierarchical logistic regression model at a tertiary care hospital in Ethiopia.

## 2 Methods and Participants

### 2.1 Study Design, Setting, and Participants

An institution‐based cross‐sectional study was conducted from August to November 2023 at Jimma University Medical Center (JUMC) located 356 km southwest of Addis Ababa, the capital city of Ethiopia. JUMC is the only teaching and referral hospital in the southwestern part of the country. It provides healthcare services through 16 departments, serving approximately 15,000 inpatients, 160,000 outpatients, 11,000 emergency cases, and 4500 deliveries annually, from a catchment population of nearly 15 million people [36, 37]. All healthcare professionals including medical doctors (with or without subspecialty/specialty), dental doctors (with or without subspecialty/specialty), resident physicians, nurses, midwives, and pharmacists working across the seven departments of JUMC were eligible to participate in the study. Healthcare professionals who were on maternity or annual leave during the study period, as well as those who declined of consent, were excluded.

### 2.2 Sample Size Calculation and Sampling Techniques

The minimum sample size was calculated using a single population proportional formula, assuming a proportion of good knowledge (55.4%) among healthcare professionals toward infection prevention measures from a previous study conducted in Addis Ababa [38], a 5% level of significance (*Zα*/2 = 1.96), a 3% margin of error, and a 10% nonresponse rate. Based on these assumptions, the estimated sample size was 328. For the participant recruitment, the seven departments: internal medicine, surgery, orthopedic and traumatology, gynecology and obstetrics, pediatrics, oncology, and dental medicine were randomly selected from JUMC clinical departments. Proportional allocation was then applied based on the number of healthcare professionals in each department, and participants were selected using a simple random sampling technique.

### 2.3 Data Collection Tool and Procedures

Data were collected using structured, self‐administered questionnaires, prepared in English after thorough review of the literature [11, 25, 39, 40], and pretested prior to actual data collection. The questionnaires consisted of four sections: sociodemographic and clinical experiences factors, knowledge domain (13 items assessing IPC Knowledge), attitude domain (6 items assessing IPC attitudes), and practices domain (10 items assessing IPC practices). Seven Bachelor of Science (BSc) nurses served as data collectors under the supervision of two Master of Science (MSc) healthcare professionals. Both data collectors and supervisors received training on the data collection tool and procedures. Daily supervision was conducted by the supervisors and principal investigators to ensure completeness and accuracy of the data. Internal reliability analyses were performed for the KAP assessment tools. Cronbach's *α* demonstrated acceptable reliability for the attitude (*α* = 0.70) and practice (*α* = 0.73) domains, while the Kuder–Richardson formula 20 (KR‐20) indicated acceptable reliability for the knowledge domain (KR‐20 = 0.76).

### 2.4 Variables and Operational Definitions

The outcome variables were knowledge (good/poor), attitude (favorable/unfavorable), and practices (safe/unsafe) of healthcare professionals toward IPC, whereas sociodemographic characteristics, year of services, weekly working hours, department, availability of IPC committee and guidelines at departments, and IPC training were independent variables. Knowledge status of healthcare professionals computed from 13 yes or no questions. Correct responses scored 1; incorrect responses scored 0, which made 13 the maximum possible score in the knowledge domain.

Regarding the attitude domain, computed from six questions, each question had three response categories: agree, neutral, or disagree. Each correct response to question scored 1, whereas neutral or incorrect responses scored 0. Practice was assessed using 10 Likert‐scale questions across four domains, including PPE use, hand hygiene, waste management, and equipment disinfection. The responses ranged from “always” (Score 5) to “never” (Score 1).

Scores for KAP were summed and converted into a 100‐point scale. A cutoff of ≥ 70% was used to classify good knowledge, favorable attitude, and safe practice; scores < 70% were categorized as poor knowledge, unfavorable attitude, or unsafe practice [32].

### 2.5 Data Processing and Analysis

**Figure 1:**
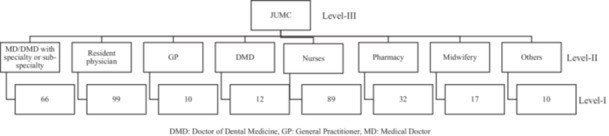
Nested structure of the health professionals, their professional, and hospital at JUMC, Ethiopia, 2023.

The collected data were entered into EpiData version 3.1 and subsequently exported to the STATA version 17 for analysis. Dependent variables were categorization, and explanatory data analysis was performed to ensure the most suitable model. Descriptive statistics were used to summarize the data: frequencies and percentages for categorical variables, whereas means with standard deviations (SD) for continuous variables. Because responses from professionals within the same discipline were correlated, healthcare professionals were considered as nested within their professions (Figure 1). The intracluster correlation coefficient (ICC) was > 0 (ICC = 0.079, 95% CI: 0.01, 0.44), and the likelihood ratio test indicated a significant difference between hierarchical logistic models with and without random effects (*χ* ^2^ = 4.57, *p* = 0.03). Accordingly, hierarchical logistic regression was selected over standard logistic regression to identify predictors of good knowledge, favorable attitudes, and safe practices. Prior to model fitting, assumptions of binary logistic regression were checked. Adjusted odds ratios (AOR) with corresponding 95% confidence intervals (CI) were computed to assess the strength and significance of the association between dependent and independent variables. The statistical tests were two‐sided, and a *p* value < 0.05 was considered statistically significant. Data management and analyses were performed in STATA version 17.0 using the built‐in *melogit* command.

### 2.6 Ethics Approval and Consent to Participate

Ethical approval had been obtained from the Institutional Review Board (IRB) of the Institute of Health, Jimma University, before the study commenced (Ref. No.: JUIH/IRB/542/23). Written informed consent was obtained from each participant after a clear explanation of the study objectives and purpose. This study was conducted in accordance with the Declaration of Helsinki.

## 3 Results

### 3.1 Sociodemographic Characteristics and Clinical Experiences of Study Participants

A total of 328 healthcare professionals participated in this study, yielding a 100% response rate. Participants were drawn from Internal Medicine (23.5%), Gynecology and Obstetrics (20.7%), Surgery (17.1%), Pediatrics (15.9%), Oncology (8.8%), Dental Medicine (8.2%), and Orthopedics (5.5%). The mean age of study participants was 31.25 years (SD ± 5.15). By profession, 92 (28.0%) were resident physicians, 89 (27.1%) nurses, 66 (20.1%) medical doctors (MD) or doctors of dental medicine (DMD) with specialty/subspecialty physician, 32 (9.8%) pharmacists, 17 (5.2%) midwives, 10 (3.0%) general practitioners (GPs), 12 (3.7%) DMDs, and 10 (3.0%) were categorized as “others” (including porters, physicists, and cleaners). Nearly three‐fourths (73.2%) of participants were males, and more than half (58.5%) were married. Regarding education, 114 (34.8%) held a BSc degree, 113 (34.5%) an MD/DMD degree, and 149 (45.4%) had 6–10 years of service experience. Only 132 (40.2%) had ever received IPC training (Table 1).

**Table 1:** Sociodemographic characteristics and experiences of health professionals regarding infection prevention at JUMC, Oromia, Ethiopia, 2023 (*n* = 328).

Variables	Category	*N* (%)
Age in years (mean ± SD)		31.25 ± 5.15
Sex	Male	240 (73.2)
	Female	88 (26.8)
Marital status	Single	130 (39.6)
	Married	192 (58.5)
	Widowed/divorced	6 (1.8)
Educational level	Diploma	8 (2.4)
	BSc	114 (34.8)
	MD/DMD	113 (34.5)
	MSc degree	27 (8.2)
	Specialty/subspecialty	66 (20.1)
Profession/field of study	MD/DMD with specialty/subspecialty	66 (20.1)
	Resident physician	92 (28.0)
	GPs	10 (3.0)
	DMD	12 (3.7)
	Nurses	89 (27.1)
	Midwives	17 (5.2)
	Pharmacists	32 (9.8)
	Other^a^	10 (3.0)
Department	Internal medicine	77 (23.5)
	Oncology	29 (8.8)
	Surgery	56 (17.1)
	Gynecology/obstetrics	68 (20.7)
	Pediatrics	52 (15.9)
	Dental medicine	27 (8.2)
	Orthopedics	18 (5.5)
Years of service	< 5 years	138 (42.1)
	6–10 years	149 (45.4)
	> 10 years	41 (12.5)
Ever taken IPC training	Yes	132 (40.2)
	No	196 (59.8)
Availability of IPC guidelines in the working department	Yes	201 (61.3)
	No	127 (38.7)
Availability of IPC committee in the working department	Yes	212 (64.6)
	No	116 (35.4)
Working hours per week	≤ 40 h	32 (9.8)
	> 40 h	296(90.2)
HCWs perception on their working load	High	206 (62.6)
	Medium	100 (30.5)
	Low	22 (6.7)

Abbreviations: BSc, Bachelor of Science; DMD, Doctor of Dental Medicine; GPs, general practitioners; HCWs, healthcare workers; IPC, infection prevention and control; MD, medical doctor; MSc, Master of Science.

^a^ Cleaners, porters, physicists.

### 3.2 The Overall KAP Status of Healthcare Professionals Toward IPC

**Figure 2:**
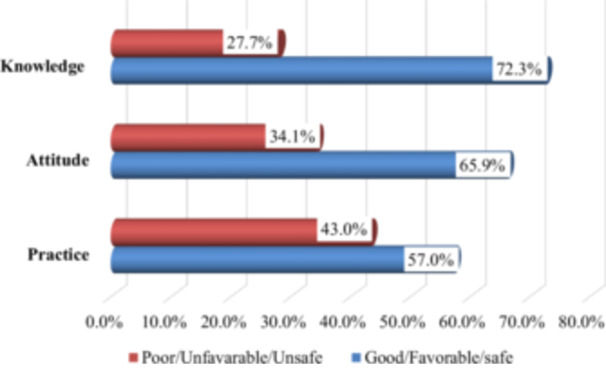
The overall scores of knowledge, attitude, and practice of health professionals regarding IPC at JUMC, Oromia, Ethiopia, 2023 (*n* = 328).

Among the 328 participants, 72.3% [95% CI: 67.0%, 77.0%] demonstrated good knowledge of IPC, 65.9% [95% CI: 61.0%, 71.0%] had a favorable attitude, and 57.0% [95% CI: 52.0%, 62.0%] reported safe IPC practices. The overall IPC KAP scores are summarized in Figure 2.

Almost all participants (97.0%) recognized that handwashing with soap or sanitizer reduces infection transmission. However, less than one‐fifth (18.9%) correctly identified that a hand sanitizer is less effective than soap and water if hands are not visibly dirty (Table 2).

**Table 2:** The health professionals response to knowledge questions regarding IPC at JUMC, Oromia, Ethiopia, 2023 (*n* = 328).

Knowledge evaluating questions	Correct response *N* (%)	Incorrect response *N* (%)
Washing hands with soap or hand sanitizer decreases the risk of transmission of infections	318 (97.0)	10 (3.0)
Use of a hand sanitizer for hand hygiene is as effective as soap and water if hands are not visibly dirty	62 (18.9)	266 (81.1)
All medical equipment/devices require disinfection after use	262 (79.9)	66 (20.1)
There is no need to wash hands prior to patient contact, if hands are not visibly dirty	253 (77.1)	75 (22.9)
Gloves provide complete protection against acquiring or transmitting infection	124 (37.8)	204 (62.2)
Wearing the same pair of gloves is possible for multiple patients as long as there is no visible contamination	240 (73.2)	88 (26.8)
High‐touch surfaces should be cleaned more frequently than low‐touch surfaces to reduce healthcare‐associated infections	276 (84.1)	52 (15.9)
Medical waste bins must be filled no more than 2/3rd full	265 (80.8)	63 (19.2)
Isolation and cohorting are only needed in outbreak situations	214 (65.2)	114 (34.8)
Overcrowded working area increases transmission of Infection	316 (96.3)	12 (3.7)
Used sharps must be discarded in puncture‐proof containers	299 (91.2)	29 (8.8)
Wound dressing can be disposed in an ordinary garbage bin	150 (45.7)	178 (54.3)
Following standard operation procedures decreases the risk of contamination	319 (97.3)	9 (2.7)
Over all knowledge	237 (72.3)	91 (27.7)

A majority (81.7%) agreed that hospitals should have a dedicated IPC committee, while only 30.2% identified resources scarcity as the main barrier to sustaining IPC practices (Table 3).

**Table 3:** The health professionals response to attitude questions regarding IPC at JUMC, Oromia, Ethiopia, 2023 (*n* = 328).

Attitude evaluating questions	Agree *N* (%)	Disagree *N* (%)	Neutral *N* (%)
It is compulsory for hospitals to have a dedicated IPC committee for infection control	268 (81.7)	38 (11.6)	22 (6.7)
I feel that the infection control policies and guidelines are sufficient in the hospital	96 (29.3)	198 (60.7)	34 (10.4)
The workload affects my ability to follow infection prevention guidelines	226 (68.9)	87 (26.5)	15 (4.6)
Scarcity of the resources are the major barrier to sustain infection prevention practices in this hospital	99 (30.2)	202 (61.6)	27 (8.2)
Practicing adequate personal hygiene decreases the risk of contamination	317 (96.6)	5 (1.5)	6 (1.8)
Needle stick or sharp injuries should be immediately documented and reported to the higher authority	258 (78.7)	35 (10.7)	35 (10.7)

Regarding practice, only 22.0% reported always washing their hands before and after patients contact, and 21.0% reported always washing their hands before leaving the hospital. Alarmingly, 97 (29.6%) indicated that they rarely or never disinfect medical equipment with disinfectants such as 10% sodium hypochlorite or bleach (Table 4).

**Table 4:** The health professionals response to practice questions regarding IPC at JUMC, Oromia, Ethiopia, 2023 (*n* = 328).

Practices enquiries questions	Always *N* (%)	Often *N* (%)	Sometimes *N* (%)	Rarely *N* (%)	Not at all *N* (%)
How often do you wash your hands before and after contact with patients?	72 (22.0)	65 (19.8)	123 (37.5)	52 (15.9)	16 (4.9)
How often do you wash your hands before leaving the hospital?	69 (21.0)	80 (24.4)	117 (35.7)	49 (14.9)	13 (4.0)
How often do you wear gloves when performing invasive procedures (cannula, catheter, etc.)?	209 (63.7)	49 (14.9)	40 (12.2)	9 (2.7)	21 (6.4)
How often do you dispose of sharp instruments immediately after use in a puncture‐proof container?	192 (58.5)	80 (24.4)	23 (7.0)	15 (4.6)	18 (5.5)
How often do you dispose used gloves and other items in the proper place?	177 (54.0)	98 (29.9)	29 (8.8)	14 (4.3)	10 (3.0)
How often do you change gloves between patients irrespective of a patient's infectious status?	167 (50.9)	74 (22.6)	47 (14.3)	14 (4.3)	26 (7.9)
How often do you wear a gown/apron if soiling with blood or body fluids is likely?	152 (46.3)	65 (19.8)	43 (13.1)	25 (7.6)	43 (13.1)
How often do you wear a disposable facemask/face shield whenever there is a possibility of a splash or splatter?	122 (37.2)	82 (25.0)	64 (19.5)	26 (7.9)	34 (10.4)
How often do you disinfect medical equipment/devices with disinfectants (such as 10% sodium hypochlorite or bleach)	94 (28.7)	68 (20.7)	69 (21.0)	37 (11.3)	60 (18.3)
How often do you inform authorized body about patients with highly transmissible infection (e.g., COVID‐19, measles, TB, etc.) for isolation	253 (77.1)	0	0	0	49 (14.9)

### 3.3 The Predictors of Good Knowledge Toward IPC

Multivariable hierarchical binary logistic regression analysis identified IPC training, availability of IPC guidelines, and years of service as significant predictors of good knowledge. Healthcare professionals who had received IPC training were more than two times more likely to demonstrate good knowledge of IPC compared to those who had not received IPC training (AOR: 2.31; 95% CI: 1.23, 4.35; *p* = 0.009). Likewise, professionals working in departments with IPC guidelines were nearly six times more likely to possess good knowledge than those in department lacking such guidelines (AOR: 5.87; 95% CI: 2.08, 16.44; *p* = 0.001). Furthermore, healthcare professionals with ≥ 10 years of service were more than three times as likely to have good knowledge of IPC compared to those with ≤ 5 years of service (AOR: 3.56; 95% CI: 1.28, 9.68; *p* = 0.01) (Table 5).

**Table 5:** Predictors of health professions' good knowledge regarding IPC at JUMC, Oromia, Ethiopia, 2023 (*n* = 328).

Variables	Category	Knowledge status		COR (95% CI)	AOR (95% CI)	*p*
		Good	Poor			
Working hours per week	≤ 40 h	19	13	0.56 (0.26, 1.22)*	0.67 (0.28, 1.59)	0.37
	> 40 h	218	78	1	1	
Ever taken IPC training	Yes	112	20	3.32 (1.88, 5.93)***	**2.31 (1.23, 4.35)** **	**0.009**
	No	125	71	1	1	
Availability of IPC committee in the working department	Yes	170	42	2.97 (1.77, 4.90)***	0.49 (0.17, 1.40)	0.19
	No	67	49	1	1	
Availability of IPC guidelines in the working department	Yes	168	33	4.31 (2.56, 7.24)***	**5.87 (2.08, 16.44)** ***	**0.001**
	No	69	56	1	1	
Service years	≤ 5 yrs	84	54	1	1	
	6–10 yrs	118	31	2.69 (1.55, 4.66)***	**2.61 (1.43, 4.71)** **	**0.002**
	≥ 10 yrs	35	6	4.01 (1.52, 10.49)**	**3.56 (1.28, 9.68)** **	**0.01**

*Note:* 1 = reference. Bold values indicate statistically significant.

Abbreviations: AOR, adjusted odds ratio; CI, confidence interval; COR, crude odds ratio.

* *p* < 0.25;

** *p* < 0.05;

*** *p* < 0.001.

### 3.4 The Predictors of Favorable Attitude Toward IPC

After adjusting for potential confounders, healthcare professionals who had received training in IPC were 80% more likely to exhibit a favorable attitude toward IPC compared to those without such training (AOR, 1.80; 95% CI, 1.08, 2.98; *p* = 0.03). Similarly, healthcare professionals working in department equipped with IPC guidelines were more than twice likely demonstrate a favorable attitude toward IPC than those in department lacking IPC guidelines (AOR, 2.14; 95% CI, 1.34, 3.42; *p* = 0.002) (Table 6).

**Table 6:** Predictors of health professionals' favorable attitude toward IPC at JUMC, Oromia, Ethiopia, 2023 (*n* = 328).

Variables	Category	Attitude		COR (95% CI)	AOR (95% CI)	*p*
		Favorable	Unfavorable			
Sex	Male	167	73	0.56 (0.26, 1.22)*	1.42 (0.85, 2.38)	0.18
	Female	49	39	1	1	
Ever taken IPC training	Yes	97	35	1.79 (1.11, 2.90)**	**1.80 (1.08, 2.98)** **	**0.03**
	No	119	77	1	1	
Availability of IPC committee in the working department	Yes	150	62	1.83 (1.14, 2.93)**	0.71 (0.28, 1.80)	0.48
	No	66	50	1	1	
Availability of IPC guidelines in the working department	Yes	146	55	2.16 (1.36, 3.44)**	**2.14 (1.34, 3.42)** **	**0.002**
	No	70	57	1	1	

*Note:* 1 = reference. Bold values indicate statistically significant.

Abbreviations: AOR, adjusted odds ratio; CI, confidence interval; COR, crude odds ratio.

* *p* < 0.25;

** *p* < 0.05.

### 3.5 The Predictors of Safe Practices

Safe practices were significantly associated with training, guideline availability, years of services, and infrastructure. Healthcare professionals who had received IPC training were 75% more likely to report safe IPC practices than those without such training (AOR, 1.75; 95% CI, 1.06, 2.89; *p* = 0.03). Those working in departments where IPC guidelines were available were nearly three times more likely to report safe IPC practices compared to those in departments lacking such guidelines (AOR, 2.65; 95% CI, 1.62, 4.36; *p* < 0.001). Moreover, professionals with 6–10 years of service were 72% more likely to exhibit safe practices than those with ≤ 5 years of service (AOR, 1.72; 95% CI, 1.04, 2.84; *p* = 0.03). In addition, professionals working in departments with handwashing facilities and water available were 99% more likely to demonstrate safe practices than those in departments lacking such facilities (AOR, 1.99; 95% CI, 1.24, 3.21; *p* = 0.005) (Table 7).

**Table 7:** Predictors of health professionals' safe practice of IPC at JUMC, Oromia, Ethiopia, 2023 (*n* = 328).

Variables	Category	Practice		COR (95% CI)	AOR (95% CI)	*p*
		Good	Poor			
Sex	Male	144	96	1.57 (0.96, 2.57)*	1.45 (0.85, 2.46)	0.17
	Female	43	45	1	1	
Working loads	≤ 40 h/week	17	15	0.64 (0.31, 1.32)*	0.82 (0.38, 1.81)	0.63
	> 40 h/week	172	124	1	1	
Ever taken IPC training	Yes	91	41	2.31 (1.46, 3.67)***	**1.75 (1.06, 2.89)** **	**0.03**
	No	96	100	1	1	
Availability of IPC committee in the working department	Yes	139	73	2.70 (1.69, 4.30)***	1.07 (0.43, 2.48)	0.89
	No	48	68	1	1	
Availability of IPC guidelines in the working department	Yes	136	45	3.12 (1.97, 4.95)***	**2.65 (1.62, 4.36)** ***	**< 0.001**
	No	51	76	1	1	
Service years	≤ 5 yrs	68	70	1	1	
	6–10 yrs	95	54	1.81 (1.13, 2.90)**	**1.72 (1.04, 2.84)** **	**0.03**
	≥ 10 yrs	24	17	1.45 (0.72, 2.94)	1.11 (0.52, 2.34)	0.79
Handwashing facilities and water availability in the working department	Yes	100	53	1.91 (1.22, 2.98)**	**1.99 (1.24, 3.21)** **	**0.005**
	No	87	88	1	1	

*Note:* 1 = reference. Bold values indicate statistically significant.

Abbreviations: AOR, adjusted odds ratio; CI, confidence interval; COR, crude odds ratio.

* *p* < 0.25;

** *p* < 0.05;

*** *p* < 0.001.

## 4 Discussion

The present study revealed that 72.3% [95% CI: 67.0%, 77.0%] of healthcare professionals demonstrated good knowledge of infection prevention. This finding is comparable with results from Addis Ababa (69%) [30] and Gondar (77.3%) [41]. However, the proportion was higher than reports from other Ethiopian studies [42, 43, 44] and from Botswana [45], but lower than that reported in Bahir Dar (84.5%) [46]. These variations may be explained by difference in knowledge scoring methods, level of training, and work experience among healthcare professionals. In the previous studies, knowledge was categorized as good or poor knowledge based on whether scores were above or below the mean. Additionally, methodological differences may account for the variation, as some studies assessed only specific aspects of infection prevention such as hand hygiene compliance or tuberculosis infection control.

The current study was conducted at a tertiary care hospital where advanced medical procedures are usually performed and large amounts of infectious waste are generated. Despite this, more than one‐fourth of the healthcare professionals were found to have poor knowledge of infection prevention. Major knowledge gaps were identified biomedical waste disposal, disinfection of medical equipment after use, handwashing prior to patient contacts, and isolation of infectious patients. These findings are consistent with reports from many hospitals in LMICs [3, 47, 48], where prevention of HCAIs receives insufficient attention. At this medical care, only 40.2% of healthcare professionals had received training on infection prevention. The lack of training and limited availability of IPC guidelines across clinical units may explain the poor knowledge observed among some of professionals.

It is well recognized that healthcare professionals' favorable attitude toward infection prevention is fundamental to reducing HCAIs [11, 39]. In the present study, about two‐thirds (65.9% [95% CI: 61.0%, 71.0%]) of healthcare professionals demonstrated a favorable attitude. This finding corroborates a study conducted at the University of Gondar referral hospital, which reported that 64.2% of healthcare professionals had a favorable attitude [33]. On the other hand, the present study's finding is inconsistent with the earlier study reported, which reported that only 55.6% of healthcare professionals had a favorable attitude [38]. This difference may be attributed to variations in participants' academic background, sampling approaches, and study settings. For instance, previous studies did not consider specialties and subspecialties and were conducted in a primary healthcare unit, which may have contributed to the lower proportion of favorable attitudes observed. A positive attitude is a key initiator of change among healthcare professionals toward effective IPC practices. Therefore, the medical center should organize regular training and workshops to refresh and update knowledge and to strengthen positive attitudes toward infection prevention.

Despite the good knowledge documented among the majority of healthcare professionals, only 57.0% [95% CI: 52.0%, 62.0%] demonstrated safe infection prevention practices in this study. The substantially lower level of practice, despite good knowledge, may be explained by the impact of high effort–reward imbalance (ERI), which often leads to burnout among professionals, and is common in large healthcare facilities such as JUMC [49, 50]. Even though, adherence to infection prevention procedures is fundamental to ensuring quality of care, and critical in preventing HCAIs [22, 23], the negative impact of ERI‐induced burnout contributes to unsafe practices of these essential procedures [51].

The lower proportion of safe IPC practices observed in this study is consistent with several studies conducted in Ethiopia [32, 33, 46, 52, 53]. Much lower levels of practices were reported in Gondar (36%) [25], and Mekele (42.9%) [54]. In the current study, only 22% of healthcare professionals reported washing their hands before and after patients contact, and 21% washed their hands before leaving the hospital. The limited availability of properly functioning sanitary facilities and the lack of continuous water supply may explain the poor handwashing practices observed. Furthermore, noncompliance with the appropriate use and disposal of PPE was another significant gap identified, corroborating findings from a study conducted in the public hospitals in northeastern Ethiopia [53]. Other studies also support these results, consistently revealing poor hand hygiene and PPE utilization among healthcare professionals [55, 56, 57].

In the current study, education and training of healthcare professionals, along with the provision of IPC guidelines at clinical departments, were significantly associated with good knowledge, favorable attitudes, and safe IPC practices. These findings are consistent with reports from Ethiopia [31, 32, 38, 42, 46, 47], Italy [58], Nepal [59], and Nigeria [40]. Healthcare professionals who had received any form of IPC training were more likely to demonstrate better KAP toward IPC. Similarly, increased KAP were observed among healthcare professionals working in clinical wards where IPC guidelines were available. The presence of IPC guidelines in clinical wards provides updated information, which may enhance professionals' KAP. These findings highlight the importance of regular training and the provision of IPC guidelines in improving healthcare professionals' KAP, eventually contributing to better infection prevention.

Interestingly, the present study revealed a clear significant association between good knowledge and years of service, indicating that longer working experience is linked to better knowledge of infection prevention. This finding is consistent with studies conducted by Geberemariyam et al. [43] and Assefa et al. [32]. Healthcare professionals with more years of service are more likely to be exposed to infection prevention information and gain experience through alongside senior staff, which could potentially enhance both their knowledge and practice. As expected, the availability of fully functioning handwashing facilities and sufficient water in clinical departments was identified as an independent factor associated with good infection prevention practices among healthcare professionals. This result is also in line with findings reported by Geberemariyam et al. [43], Bayleyegn et al. [25], and Assefa et al. [32].

Despite the valuable findings, this study has several limitations. First, due to its cross‐sectional design, it is impossible to establish a temporal relationship between dependent and independent variables. Second, recall bias among healthcare professionals may have influenced the accuracy of the responses. Finally, since the study was conducted at a tertiary care hospital, the findings may not be generalizable to primary and secondary care hospitals. Nevertheless, the present study also has notable strengths. It accounted for the dependence of responses by applying hierarchical logistical regression, included physicians with specialties and subspecialties, assessed healthcare professionals' KAP in the post‐COVID‐19 pandemic period, and achieved a 100% response rate, thereby enhancing the robustness and creditability of the results.

## 5 Conclusion

The present study highlighted that the majority of healthcare professionals have adequate knowledge and a favorable attitude toward infection prevention. However, the overall proportion of safe infection prevention practices among healthcare professionals in the study setting remains suboptimal. Training on infection prevention and the availability of IPC guidelines in the workplace were found to be predictors of good knowledge, favorable attitudes, and safe practices. Therefore, hospital authorities and IPC committee members should organize regular on‐the‐job training for healthcare professionals and ensure that IPC guidelines are available in every clinical ward. In addition, hospital management should guarantee the presence of fully functioning handwashing facilities, a continuous water supply, and PPE to reduce the risks of HCAIs. The provision of well‐functioning sanitary facilities and sufficient water and appropriate PPE such as gloves and facemasks is crucial to improving safe IPC practices among healthcare professionals. We strongly recommend that hospital administrators and facility managers ensure that handwashing facilities are consistently operational and that water is available in sufficient quantities at all times to strengthen safe infection prevention practices and thereby reduce HCAIs and ARM.

## Author Contributions

Abebe Dukessa Dubiwak and Mulualem Tadesse conceptualized and wrote the main manuscript. Belay Zewdie, Abebaw Tiruneh, Selam Tesfaye, and Amare Assefa reviewed and edited the manuscript. Tadele Akeba Diriba, Gemeda Abebe, and Lelisa Sena Dadi reviewed and edited the manuscript and supervised the overall data analysis and visualization. All authors have read and approved the final version of the manuscript. Abebe Dukessa Dubiwak had full access to all of the data in this study and takes complete responsibility for the integrity of the data and the accuracy of the data analysis.

## Funding

The authors received no specific funding for this work.

## Disclosure

The lead author Abebe Dukessa Dubiwak affirms that this manuscript is an honest, accurate, and transparent account of the study being reported; that no important aspects of the study have been omitted; and that any discrepancies from the study as planned (and, if relevant, registered) have been explained.

## Ethics Statement

Ethical approval had been obtained from the Institutional Review Board (IRB) of the Institute of Health, Jimma University, before the study commenced (Ref. No.: JUIH/IRB/542/23). This study was conducted in accordance with the Declaration of Helsinki.

## Consent

Written informed consent was obtained from each participant after a clear explanation of the study objectives and purpose.

## Conflicts of Interest

The authors declare no conflicts of interest.

## Data Availability

The data supporting the findings of this study are available from the corresponding author upon reasonable request.
